# Pregabalin versus gabapentin in the management of peripheral neuropathic pain associated with post-herpetic neuralgia and diabetic neuropathy: a cost effectiveness analysis for the Greek healthcare setting

**DOI:** 10.1186/1471-2377-13-56

**Published:** 2013-06-04

**Authors:** Kostas Athanasakis, Ioannis Petrakis, Eleftheria Karampli, Elli Vitsou, Leonidas Lyras, John Kyriopoulos

**Affiliations:** 1 Department of Health Economics, National School of Public Health, Athens, Greece; 2 Pfizer Hellas, Athens, Greece

**Keywords:** Peripheral neuropathic pain, Post-herpetic neuralgia, Diabetic neuropathy, Pregabalin, Gabapentin, Cost-effectiveness analysis

## Abstract

**Background:**

The anticonvulsants pregabalin and gabapentin are both indicated for the treatment of peripheral neuropathic pain. The decision on which treatment provides the best alternative, should take into account all aspects of costs and outcomes associated with the two therapeutic options. The objective of this study was to examine the cost – effectiveness of the two agents in the management of patients with painful diabetic neuropathy or post – herpetic neuralgia, under the third party payer perspective in Greece.

**Methods:**

The analysis was based on a dynamic simulation model which estimated and compared the costs and outcomes of pregabalin and gabapentin in a hypothetical cohort of 1,000 patients suffering from painful Diabetic Peripheral Neuropathy (DPN) or Post-Herpetic Neuralgia (PHN). In the model, each patient was randomly allocated an average pretreatment pain score, measured using an eleven-point visual analogue scale (0 – 10) and was “run through” the model, simulating their daily pain intensity and allowing for stochastic calculation of outcomes, taking into account medical interventions and the effectiveness of each treatment.

**Results:**

Pregabalin demonstrated a reduction in days with moderate to severe pain when compared to gabapentin. During the 12 weeks the pregabalin arm demonstrated a 0.1178 (SE 0.0002) QALY gain, which proved to be 0.0063 (SE 0.0003) higher than that in the gabapentin arm. The mean medication cost per patient was higher for the pregabalin arm when compared to the gabapentin arm (i.e. €134.40) over the 12 week treatment period. However, this higher cost was partially offset by the reduced direct medical costs (i.e. the cost of specialist visits, the cost of diagnostic tests and the other applied interventions). Comparing costs with respective outcomes, the ICERs for pregabalin versus gabapentin were €13 (95%CI: 8 – 18) per additional day with no or mild pain and €19,320 (95%CI: 11,743 – 26,755) per QALY gained.

**Conclusions:**

Neuropathic pain carries a great disease burden for patients and society and, is also, associated with a significant economic burden. The treatment of pain associated with DPN and PHN with pregabalin is a cost-effective intervention for the social security in Greece compared to gabapentin. Thus, these findings need to be taken into consideration in the decision – making process when considering which therapy to use for the treatment of neuropathic pain.

## Background

Neuropathic pain (NeP) is defined by the International Association for the Study of Pain (IASP) as “Pain caused by a lesion or disease of the somatosensory nervous system”. NeP can be a result of a variety of conditions associated with impairing the functioning of the nervous system, such as diabetes, multiple sclerosis, trauma and herpes zoster infections [1]. It is a common condition with an overall prevalence between 0.9 and 8.0% [1,2]. Previous literature suggests that individuals with NeP were known to experience more severe pain when compared to non-NeP chronic pain sufferers [1]. Despite the plethora of etiologies associated with NeP, the scientific focus lies mainly on painful diabetic peripheral neuropathy (DPN) and post-herpetic neuralgia (PHN), extrapolating any outcomes on other causes of NeP [3]. Painful DPN is a common complication of diabetes with a prevalence of up to 25% among diabetic patients [3]. PHN is in turn the most common chronic complication of herpes zoster infection (10 – 75% of cases) [4,5]. Neuropathic pain has been associated with impaired quality of life, reduced individual productivity and increased patient and healthcare resource expenditure [3,6]. Co-morbid conditions include sleep disturbances, depression and anxiety disorders [6], increasing even further the economic burden to the healthcare system. In a recent review, the average pain severity associated with painful DPN and PHN was identified to be 5.0/10 and 4.4/10 (Visual Analog Scale) and the average EQ-5D values, for patients with severe pain, equal to 0.2 and 0.26 respectively [3].

The anticonvulsants pregabalin and gabapentin are indicated for the treatment of neuropathic pain. Treatment with the third generation anticonvulsant - pregabalin can be started at a dose of 150 mg per day given as two to three divided doses. Based on individual patient response and tolerability, the dose may be gradually increased, if needed, to a maximum dose of 600 mg per day. Clinical trials using pregabalin for both peripheral and central NeP, showed a reduction in pain scores within the first week, which was maintained throughout the treatment period [7,8]. Alternatively, the starting dose of gabapentin is 900 mg/day given as three equally divided doses, increasing gradually up to a maximum daily dose of 3,600 mg. Clinical trials have shown that the optimal daily dosing for pain control exceeded 1,800 mg [9,10].

A cohort study by Toth et al. [11] investigated the utility associated with the substitution of gabapentin with pregabalin therapy in patients with peripheral NeP. Results showed that both previous responders and non-responders to gabapentin had additional pain relief of approximately 25%, six or twelve months after initiation of pregabalin. Another study by Tarride et al. showed that following a twelve-week regime, therapy with pregabalin was associated with nine additional days with no or mild pain, against six additional days with gabapentin therapy [11].

Along with the previously mentioned high incidence, chronicity, maladaptivity and co-morbidities associated with NeP, comes the significant economic burden to the national health system. In an attempt to estimate the costs associated with NeP, Dworkin et al. (2010) calculated the excess healthcare costs associated with peripheral NeP between $1,600 and $7,000 [12]. In the same context, Berger and colleagues estimated [13], that the excess expenditure of patients with NeP, can reach a threefold increase compared to their non-NeP peers ($17,355 versus $5,715, 2000 values). When investigating costs associated with painful DPN, Gordois et al. found that direct medical costs exceeded $10billion per year in the United States [14]. Another study, found that the average medical costs due to PHN following herpes zoster infections ranged from $760 to $1300 per patient for the first year after infection (2004 values) [15]. Apart from the direct costs mentioned above, another dimension of costs, the societal costs from NeP also need to be taken into account. Characteristically, in a cross-sectional European study, researchers identified that 43% of patients reported work absence and even change in employment status and 17% were disabled due to NeP [16].

Thus, the benefit of treatment for patients with chronic neuropathic pain is dual, including both the effects of reduced morbidity as well as their subsequent contribution in societal and health care costs. However, the decision on which treatment provides the best alternative, should take into account all aspects of treatment costs included. In this decision – making process, pharmacoeconomic tools, such as economic evaluation, are deemed pivotal. In light of the above, the purpose of this study was to examine the cost – effectiveness of pregabalin versus gabapentin in the management of patients with painful diabetic neuropathy or PHN in view of the third party payer in Greece*.*

## Methods

### Study model

The cost – effectiveness analysis was based on a dynamic simulation model [17,18] which estimated the costs and outcomes of pregabalin and gabapentin in a hypothetical cohort of 1,000 patients suffering from painful DPN or PHN.

**Figure 1:**
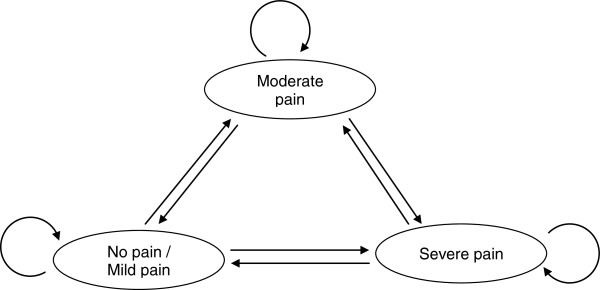
Overview of the study model.

In the model, each patient was randomly allocated an average pretreatment pain score, measured using an eleven-point visual analogue scale, with 0 referring to “no pain” and 10 to “the worst pain imaginable”, which was derived from the actual distribution of pain levels in a randomized, double-blind controlled trial of pregabalin in patients with chronic NeP, defined as subjects with DPN or PHN [7]. Following that, every patient in the cohort was “run through” the model, which used a Markovian process to simulate their daily pain intensity and allow for stochastic calculation of outcomes taking into account medical interventions and the effectiveness of each treatment. Three different health states relative to NeP were adopted from clinical practice for the purposes of the model. Specifically, days with “no or mild pain” reflected a pain intensity of “0 to < 4”, whereas days with “moderate” and “severe” pain were associated with pain scores “4 to < 7” and “7 to 10”, respectively. The randomly allocated pretreatment scores ranged from 4 to 10 (moderate to severe pain). As the patient progressed through treatment with pregabalin or gabapentin, the model projected the estimated efficacy of the two pharmacotherapies on the assigned daily pain scores, and, thus, the “journey” of patients and the respective outcomes. Default estimates, model assumptions and further description of the model have been previously presented elsewhere [3,11,19]. An outline of the model is presented in Figure 1.

The time frame of interest in the model was twelve weeks and all NeP-associated direct costs were considered. Several outcomes were derived from the above dynamic simulation model. The number of days with no or mild pain was the primary measure in the model, but also the mean number of days with 30% and 50% reduction in pain score were estimated. Other outcomes of interest included Quality Adjusted Life Years (QALYs) gained and the cost per QALY gained along with costs of medication and NeP-related healthcare services.

### Pharmacotherapies

In the model, the cost effectiveness of pregabalin at a daily dose of 150 – 600 mg (average maintenance dose of 457 mg [7]) was compared against gabapentin mean dose 2,400 mg daily (900 – 3,600 mg). The two therapies were considered to have similar side-effect profiles and therefore no discontinuation of treatment or added costs due to unwanted effects were assumed. The efficacy of the two anticonvulsants in reducing weekly pain scores (Table 1) was derived from three randomized, double-blind, controlled studies [7,9,10]. The model allows for variations in week to week reductions in pain scores in accordance to the actual distribution of change as presented in the above controlled trials.

**Table 1:** Percentage weekly change in pain severity among patients with painful DPN or PHN receiving treatment with pregabalin or gabapentin

	**Pregabalin n = 141**	**Gabapentin n = 193**
**Mean dose mg/day**	**457**	**2400**
**Mean total weekly reduction versus baseline in daily pain scores %**		
Week 1	**13.7**	**17.2**
Week 2	**23.2**	**25.1**
Week 3	**29.9**	**29.7**
Week 4	**39.1**	**32.1**
Week 5	**44.4**	**33.7**
Week 6	**44.2**	**34.9**
Week 7	**45.0**	**35.8**
Week 8	**46.3**	**36.4**
Week 9	**49.8**	**36.9**
Week 10	**51.1**	**37.2**
Week 11	**53.3**	**37.4**
Week 12	**54.1**	**37.6**

Data obtained from Protocol 1008–155 for pregabalin [7], and Protocols 945–210 and 945–211 for gabapentin [9,10].

### Healthcare resource use and medication costs

Medication costs were calculated using the latest price catalogue of medicinal products, as published in the price bulletin issued by the Ministry of Health (generic preparations of gabapentin were not included in the analysis due to their low penetration in the Greek healthcare market). Moreover, for the purposes of the analysis, it was assumed that no cost variations would result from prescribing divided doses of the comparator therapies. The costs per health service and diagnostic tools were derived from the official NHS price lists.

To identify healthcare resource utilization data according to pain severity, a survey, was conducted in a group of 100 general practitioners and 20 specialized pain clinics in Greece. General practitioners were requested to provide the percentage of patients that were referred to pain clinics, according to pain score, whereas data on utilization of diagnostic tests and other health services arose from the survey of referable specialized pain clinics (Table 2).

**Table 2:** Probability of healthcare resource use and unit costs per utilized service

**Healthcare service**		**Probability of utilization**	**Unit cost (Euros)**
**Referral to specialist**			
**Pain score**			
	0 to < 4	**0.13**	**20.00**
	4 to < 7	**0.24**	**20.00**
	7 to 10	**0.57**	**20.00**
**Diagnostic tests**			
	CAT	**0.10**	**71.11**
	MRI	**0.30**	**236.95**
	Nerve conduction studies	**0.17**	**8.63**
	Doppler sonograph	**0.12**	**27.00**
	EMG	**0.19**	**8.28**
	Blood testing (Basic Haematology Biochemistry)	**0.74**	**34.56**
	X-Ray	**0.30**	**4.05**
	γ-Ray	**0.05**	**60.16**
**Other interventions**			
	Physical therapy	**0.33**	**25.00**
	Drug infiltrations	**0.63**	**20.00**
	Nerve block	**0.39**	**14.67**
	TENS	**0.36**	**54.18**
	Spinal stimulator implant	**0.03**	**4610.90**

*CAT* – computerized axial tomography. *MRI* – magnetic resonance imaging.

*EMG* – electromyogram. *TENS* – transcutaneous electric nerve stimulation.

Physical therapy and drug infiltrations – prices per session.

The time frame of interest in the model was twelve weeks and all NeP-associated direct costs were considered, and calculated from a third party payer (social insurance) perspective and reported in year 2011 values.

### Sensitivity analyses

To address parameter uncertainty, a series of one-way sensitivity analyses, were performed, by recalculating the results, after a ±20% change in baseline values, for selected parameters. The sensitivity analysis focused on the cost of pregabalin, the weekly probability of physician visiting due to NeP and the health utility values in association with neuropathic pain. Additional scenarios of calculations included alternating daily dosages of gabapentin (1800 mg and 1200 mg) as well as the exclusion of non-medication related health-resource use (i.e. consideration of medication costs only).

## Results

The clinical outcomes at endpoint (t = 12 weeks) are presented in Table 3. Mean pretreatment pain scores were identical (6.9) for both pregabalin and gabapentin. Post-treatment pain score mean values were 4.1 for pregabalin and 4.8 for gabapentin, with the differences in the simulations being statistically significant at the 0.05 level. Pregabalin also demonstrated a statistically significant reduction in days with moderate to severe pain when compared to gabapentin. That was also apparent when measuring percentage reduction in pain scores. During the 12 weeks treatment period, the pregabalin arm demonstrated a 0.1178 (SE 0.0002) QALY gain, which proved to be 0.0063 (SE 0.0003) higher than that in the gabapentin arm (p < 0.05).

**Table 3:** Expected clinical outcomes per patient after treatment with Pregabalin or Gabapentin

	**Treatment**		
	**Pregabalin (150–600 mg/d)**	**Gabapentin (2400 mg/d)**	**Difference (Pregabalin - Gabapentin)**
**Pain score**			
Pre-treatment	**6.9 (0.0)**	**6.9 (0,0)**	**0.0 (0.0)**
Post-treatment	**4.1 (0.0)**	**4.8 (0,0)**	**-0.6 (0.0)**
**Days with**			
No or mild pain	**36 (0.3)**	**27 (0.3)**	**9 (0.5)**
Moderate pain	**32 (0.3)**	**38 (0.3)**	**-6 (0.5)**
Severe pain	**15 (0.2)**	**19 (0.3)**	**-4 (0.3)**
**Days with**			
≥30% reduction in pain score	**50 (0.3)**	**42 (0.4)**	**8 (0.5)**
≥50% reduction in pain score	**36 (0.3)**	**26 (0.4)**	**10 (0.5)**
**Quality-adjusted life-years (QALYs)**	**0.1178 (0.0002)**	**0.1115 (0.0002)**	**0.0063 (0.0003)**

Results presented as Mean (SE) for the 12 – week duration of modeling.

The mean medication cost per patient was higher for the pregabalin arm when compared to the gabapentin arm (i.e. €134.40 higher) over the 12 week treatment period. However, as presented in Table 4, this cost was partially offset by the reduced direct medical costs, such as the cost of specialist visits, the costs of diagnostic tests and the other applied interventions, which were €12 lower Comparing costs with respective outcomes, the ICERs for pregabalin versus gabapentin were €13 (95%CI: 8 – 18) per additional day with no or mild pain and €19,320 (95%CI: 11,743 – 26,755) per QALY gained. Results are summarized in Table 5.

**Table 4:** Expected medical care costs per patient

	**Treatment**		
	**Pregabalin (150–600 mg/d)**	**Gabapentin (2400 mg/d)**	**Difference (Pregabalin - Gabapentin)**
**Medication**	**306.60 (0.00)**	**172.20 (0.00)**	**134.40 (0.00)**
**Outpatient care**			
Primary care provider	**36.12 (0.70)**	**37.45 (0.74)**	**-1.33 (1.04)**
Specialist referral	**9.89 (0.33)**	**10.52 (0.33)**	**-0.63 (0.47)**
Diagnostic tests	**56.86 (3.13)**	**60.57 (2.90)**	**-3.72 (4.01)**
Other interventions	**90.88 (18.52)**	**97.22 (17.12)**	**-6.34 (24.15)**
**Total**	**500.35 (19.08)**	**377.96 (17.73)**	**122.39 (25.26)**

Values in Euros mean (SE).

For the 12 – week treatment period.

**Table 5:** Incremental cost-effectiveness ratios of pregabalin vs. gabapentin in the treatment of painful DPN and PHN

	**Pregabalin vs Gabapentin**
**Cost per additional (€)**	**Mean (95% CI)**
***Day with no or mild pain***	
Mean estimate	**13**
95% confidence interval	**(8, 18)**
**QALY gained**	
Mean estimate	**19,320**
95% confidence interval	**(11,743 - 26,755)**

The sensitivity analysis showed that parameters with the greatest impact on results were the daily cost of pregabalin and the utilities associated with pain severity (Table 6). The incremental costs per additional day with no or mild pain ranged between 7(95%CI: 2, 14) and 24 (95%CI: 18, 34), while the corresponding cost per QALY gained ranged between 11,075 (95%CI: 2,944, 23,040) and 39,073 (95%CI: 28,704, 54,620). Both values refer to a 20% lowering and a 20% rise, respectively, to the baseline daily cost of pregabalin. A 1200 mg reduction in the mean gabapentin daily dose caused the ICER value per QALY gained to exceed the €30,000 threshold.

**Table 6:** Sensitivity analysis of the incremental cost effectiveness of pregabalin versus gabapentin in the treatment of painful diabetic neuropathy and post herpetic neuralgia

**Parameter**	**Baseline**	**Sensitivity analysis**	**Cost per additional day with no or mild pain**	**Cost per QALY gained**
**Gabapentin dose (mg)**	2400	1800	**14 (8,19)**	**23 786 (14266,33498)**
**Gabapentin dose (mg)**	2400	1200	**16 (9,24)**	**30 241 (18086,44056)**
**Costs considered**	All healthcare costs	Medication cost only	**16 (14,18)**	**25 683 (22812,29829)**
**Pregabalin daily cost**	3.65	↑20%	**24 (18,34)**	**39 063 (28704,54620)**
**Pregabalin daily cost**	3.65	↓20%	**7 (2,14)**	**11 075 (2944,23040)**
**Weekly probability of PCP visit in relation to NeP**	No/mild: 0.25	↑20%	**14 (8,20)**	**21 025 (11592,30710)**
	Moderate: 0.31			
	Severe: 0.48			
**Weekly probability of PCP visit in relation to NeP**	No/mild: 0.25	↓20%	**13 (7,19)**	**19 773 (10531,28687)**
	Moderate: 0.31			
	Severe: 0.48			
**Health utility associated with pain severity**	No/mild: 0.64	↑20%	**13 (9,18)**	**17 017 (10296,23936)**
	Moderate: 0.48			
	Severe: 0.27			
**Health utility associated with pain severity**	No/mild: 0.64	↓20%	**14 (8,20)**	**27 505 (15418,40596)**
	Moderate: 0.48			
	Severe: 0.27			

Mean values in € (SE) 95% CI.

*NeP* – Neuropathic pain.

*PCP* – primary care physician.

## Discussion

The present study aimed at estimating the cost-effectiveness of pregabalin, versus gabapentin for the treatment of DPN or PHN, taking into account the perspective of a social security organization in Greece. For that purpose, a previously presented and validated health economic model was adapted for the Greek healthcare setting, taking into account direct costs of treatment and follow up for a hypothetical cohort of 1,000 patients that were treated under the two alternatives for a 12 week period, based on the efficacy profile of each intervention as recorded in the results of published clinical trials.

The results of the analysis indicate that pregabalin is a more costly but also a more effective treatment option compared to gabapentin. The excess costs of pharmaceutical treatment for pregabalin are partially offset by its improved clinical profile in terms of reductions in resource utilization and its improved outcomes in the patient level, thus leading to incremental cost-effectiveness ratios of €13 per additional day with mild/no pain and €19,320 per QALY gained.

In Greece there are currently no established thresholds under which interventions can be classified as cost-effective. In general, the accepted willingness to pay per QALY gained falls within £20,000 – £30,000 in the UK and $50,000 – $100,000 in the US [20], whereas older studies in other healthcare settings have placed this limit at a lower level (e.g. 20,000$ per QALY gained in Canada [21]). A generally acknowledged criterion (a “rule of thumb”) states that interventions costing less than 30,000€/QALY gained are “good value for money”, from an economic evaluation point of view [22], a principle that the results of the present study fulfil. Moreover the study results also meet the criteria for cost-effective interventions recommended by the WHO Commission on Macroeconomics and Health [23]. Specifically, based on the Commission’s recommendations, interventions with an ICER (expressed in cost per Disability Adjusted Life Year averted) that is lower than three times the Gross National Income (GNI) per capita can be classified as cost-effective, whereas ICERs lower than 1 × GNI indicate highly cost effective interventions. Taking into account a GNI per capita of €19,801 in Greece (2010 values) and extending the criterion to a per QALY decision, treatment of painful DPN or PHN with pregabalin falls within the range of highly cost-effective interventions.

The findings of this study are in accordance to previously published literature [3,11] that aimed to estimate the cost – effectiveness of the two pharmacotherapies. The outcomes of the Rodriguez et al. study in Spain [3] estimated an ICER (euros per QALY gained and per day with no or mild pain) of €20,535 (1,607 – 40,345) and €12 (1 – 24) respectively. The Canadian study of Tarride et al. [11] examined the two disorders separately and provided two sets of results, indicating in both cases that pregabalin was cost-effective. More specifically, regarding painful DPN, pregabalin had an ICER of $13 per day with no or mild pain and $15,708 per QALY gained respectively, whereas, for the PHN outcomes the equivalent values were $3 and $3,325, respectively (all values reported in year 2004 Canadian dollars). Moreover, the results of this study are in accordance with findings from a recent systematic review on the effectiveness and cost-effectiveness of pregabalin in the management of DPN . Meshkini et al. (2012) [24] concluded that higher doses of pregabalin (300 mg – 600 mg daily), appear highly cost-effective treatment options.

As with any study of its kind, the present one has some limitations that should be acknowledged. Firstly, the data on efficacy of the treatments under comparison are based on clinical trial data, which considered patients in a different healthcare setting than that in Greece. Thus, the trial cohorts might not be fully representative for patients with painful DPN or PHN in Greece, Nevertheless, the magnitude of this (possible) discrepancy is extremely difficult to quantify and to include in the calculations of the analysis. Moreover, the perspective of the analysis (third-party payer, i.e. the Greek Social Insurance Funds) does not include other costs, such as the indirect expenses due to productivity losses. If the societal perspective had been adopted, there is evidence that the ICERs would probably be more favourable (i.e. lower). For example, a recent cost analysis of adding pregabalin or gabapentin to the management of community – based patients with peripheral NeP, which estimated also the indirect costs, showed that although the pharmaceutical costs of pregabalin were significant, the overall patient cost was lower in the pregabalin group due to reduced sick leave and lower healthcare costs, and thus was compensated the higher treatment acquisition cost of pregabalin [25]. A limitation also arises from the fact that calculations do not include variations of cost that could arise from divided dosing regimens due to the design of the model. The same approach was used in other adaptations of the model, in different health care settings [3,11].

Another limitation of the analysis that should be considered is the source of data regarding the resource use incorporated in the calculations, i.e., the elicitation of some data via a questionnaire survey. Although an ideal approach would be to review actual patient data, the absence of centralized patient records or databases containing relevant data in the Greek NHS, rendered necessary the use of a questionnaire survey. Inevitably, the above mentioned approach introduces uncertainty in the calculations, whose extent, however, is quite difficult to quantify. Nevertheless, the magnitude of the study sample, the simplicity of the data that were requested and the extensive sensitivity analysis on the baseline values, enhance the robustness of outcomes.

Finally, the present study concludes that the intervention under investigation was followed by favourable incremental cost-effectiveness ratios, compared to other treatment strategies on pain management. However, the discussion on the adoption of such a policy by the Social Insurance will be complete, in economic terms, when accompanied by estimations of this intervention to insurance budgets, i.e. a budget impact analysis. This issue certainly constitutes an area of future research.

## Conclusion

Neuropathic pain carries a great disease burden for patients and society and, also, a significant economic burden. From a third part payer perspective, the treatment of pain associated with painful DPN and PHN with pregabalin is a cost-effective intervention for the social security in Greece compared to gabapentin. Notwithstanding its limitations, the study’s findings need to be taken into consideration in the decision – making process when considering which therapy to use for the treatment of neuropathic pain.

## Abbreviations

DPN: Diabetic peripheral neuropathy; EQ-5D: Euroqol-5D; GBP: Gabapentin; IASP: International association for the study of pain; ICER: Incremental cost effectiveness ratio; NeP: Neuropathic pain; NHS: National health service; PHN: Post herpetic neuralgia; PGB: Pregabalin; QALY: Quality adjusted life years; WHO: World health organization.

## Competing interests

EV and LL are employees of Pfizer Hellas. KA has received funding in the past from Pfizer with the purpose of data analyses and manuscript revision.

The authors declare no other financial or non-financial competing interests.

## Authors’ contribution

KA and IP performed the calculations and analyses reported in the text. EV and LL reviewed the literature for relevant data and documentation. IP and EK drafted the manuscript which was edited and critically revised by KA and JK. All authors read and approved the final manuscript.

## Pre-publication history

The pre-publication history for this paper can be accessed here:

http://www.biomedcentral.com/1471-2377/13/56/prepub
